# Statistical characteristics of the spatial distribution of wind and snowfall in the Beijing–Tianjin–Hebei Region

**DOI:** 10.1038/s41598-021-83003-8

**Published:** 2021-02-10

**Authors:** Xuetao Yu, Yajing Zhang, Qingkuan Liu, Yaya Jia, Ling Gao, Jianqing Bu

**Affiliations:** 1 State Key Laboratory of Mechanical Behavior and System Safety of Traffic Engineering Structures, Shijiazhuang Tiedao University, Shijiazhuang, 050043 People’s Republic of China; 2 Key Laboratory of Traffic Safety and Control of Hebei Province, Shijiazhuang, 050043 People’s Republic of China; 3 School of Traffic and Transportation, Shijiazhuang Tiedao University, Shijiazhuang, 050043 People’s Republic of China; 4 Wind Engineering Research Center, School of Civil Engineering, Shijiazhuang Tiedao University, Shijiazhuang, 050043 People’s Republic of China; 5 Innovation Center for Wind Engineering and Wind Energy Technology of Hebei Province, Shijiazhuang, 050043 People’s Republic of China; 6 Hebei Provincial Communications Planning and Design Institute, Shijiazhuang, 050011 People’s Republic of China

**Keywords:** Natural hazards, Environmental impact

## Abstract

In the current design specification of building structure, the basic wind pressure and basic snow pressure are two independent values, and it is impossible to acquire both of these values when snow and wind occur at the same time. Taking parameters such as snowfall intensity, snowfall amount, wind speed, and wind direction as indicators, the value of the combined distribution of wind and snowfall in the Beijing–Tianjin–Hebei region of China was extracted. A joint distribution map of the daily average snowfall among the top-ten largest consecutive snowfall events and the daily average wind scale from the first day of snowfall to the fifth day after the snowfall were obtained. The study found that after a heavy snowfall in the Zhangjiakou area, the accumulated wind power was large and, although the wind speed was favorable for the occurrence of snowdrifts, the snowfall was light. After a heavy snowfall in the Shijiazhuang area, the accumulated wind power was small, and the probability of snowdrift formation was low. In the eastern regions of Cangzhou, Beijing, Tianjin, Tangshan, and Qinhuangdao, the accumulated wind force was relatively large after a heavy snowfall, and the probability of windblown snow was relatively high.

## Introduction

When the wind speed reaches a certain intensity during or after a snowfall, the snow particles can be blown up into the air and moved with the wind, promoting the formation of snowdrifts^1^. The snow particles carried by the wind are piled up at a place where the wind speed is weakened, forming a thicker layer of snow. Importantly, snowdrifts are a major cause of snow disasters. The accumulated snow caused by snowdrifts is the material source for alpine glaciers and polar ice sheets, and this accumulation can also cause or aggravate other natural disasters, including snow and ice floods, avalanches, debris flows, landslides, and other events^2^. In some areas, the stacking thickness of snow is several times the thickness of the snowfall, causing the collapse of houses, structural damage, disruption of ground transportation, and other problems, all of which can result in severe losses to industry, transportation, agriculture, animal production, and people’s lives and property^3,4^. Moreover, with the further development of the economy, the resulting increase in the urban population, and the further development of colder regions, it is anticipated that the losses instigated by snowdrift formation to increase significantly.

Weather conditions that produce snowdrifts occur frequently on all of the continents. In December 2005, a blizzard hit Japan, causing the roofs of a gymnasium at a school in Yamagata-ken to collapse due to the weight of the snow and stagnation of most transport systems^5^. On January 3, 2010, the Inner Mongolia region of China was hit by a sudden blizzard, with the worst-hit areas experiencing snowdrifts as deep as 3.1 m. As a result, 15 carriages of the No. 1817 train from Harbin to Ulanhot were covered with snow, and more than 1400 passengers and crew were trapped on the train^6^. In December 2010, the state of Minnesota in the USA suffered from strong winds and heavy snowfall, which resulted in the closure of several state highways, the delay of a large number of flights, and the collapse of a stadium.

In view of the serious disasters caused by snowdrift formation, it is essential to study the mechanisms involved in the formation of snowdrifts. The basic parameters of snowdrift research are the probability and simultaneous occurrence of snowfall intensity, wind scale during or after the snowfall, and wind direction. In the current design specification^7^, the basic wind pressure and basic snow pressure are two independent values; however, it is not possible to acquire both of these values when snow and wind occur at the same time.

At present, the movement mechanism promoting snowdrift formation is still not entirely clear. The main research methods for such investigations include theoretical analysis, field observations, wind tunnel tests, numerical simulations, and others, with each method having its own advantages and disadvantages. In 1976, Kind made a detailed theoretical derivation and analysis concerning the saltation process of snow particles^8^, and he then detailed the critical conditions concerning the drift of particles via theoretical analysis in 1990^9^. In 2002, Tsuchiya et al*.* proposed a relationship between the accumulation thickness of snow particles and the acceleration of wind speed based on wind and snow data, which was measured on high and low roofs in Hokkaido, Japan^10^.

Doorschot et al. previously performed field measurements to calculate the threshold friction velocity for snow saltation and mass fluxes during snowdrift formation^11^, and Lü et al. conducted a series of experiments in wind tunnel to investigate the motion of natural snow (fresh snow and old snow), which was collected outdoors without altering the surface structure^12^. To simulate the dynamic process of snowfall area boundary, Fu Zhu et al. proposed an adaptive-mesh method using radial basis function (RBF) interpolation, which realized the change of the phase boundary in both two-dimensional and three-dimensional snowdrift formation^13^. Liu et al. undertook three sets of experiments to investigate the influence of wind on snowdrift accumulation around a building model and to explore its influence on two separate building models^14^. Schön et al. proposed an efficient method for generating estimations of the changes in snow heights during blowing snow events^15^.

Weiwei Zhang and Guanghui Zhang studied the formation mechanism of snowdrifts and concluded that it was largely related to severe cold weather and heavy snowfall, as well as the topography and landform of the study area, concluding that the occurrence of snowdrifts has a distinct regional character^16^. He Wu et al. studied the formation mechanism and spatial distribution characteristics of snowdrift disasters on highways^17^. Jian Liu et al. assessed the causes and main types of snowdrift situations on the roads in Xinjiang, including windward snow type, leeward snow type, snowdrifts in horizontal curves, and road cut snow type^18^. Gao et al. studied snowdrift disasters along the Jinghe–Yining railway and the control methods employed. The main control methods included setting up anti-snowdrift corridors, placing air deflectors and side guiders, and the placement of snow fencing^19^.

Generally, snowdrift formation is a joint probability event of wind and snow variables, with the two primary variables being snowfall intensity and wind strength. Only when heavy snowfall and strong winds appear together will snowdrifts be formed^20^. The above conclusion is based on the assumption that snowdrift formation will occur when there is enough snow in a certain place and the wind is strong enough during or after the snowfall event. During the snowfall event, snowdrift formation might occur if the wind blows hard enough. After a heavy snowfall, snowdrift formation might also occur before the snow surface melts and crystallizes. Therefore, it is an important basis for the assessment of snowdrift events to extract the probability of the joint distribution of snowfall and wind in a specific region from the meteorological data and to conclude the probability of the occurrence of the two parameters at the same time. Based on this, this current study assessed the joint distribution of wind and snowfall and obtained the occurrence regularity of snowdrift events in the Beijing–Tianjin–Hebei region of China using the meteorological data available from the China Meteorological Administration.

## Materials

In this section, the meteorological data used in this study is introduced. It consisted of the “Daily Data Sets of Climate Data for China International Surface Exchange Station (V3.0)”. This data was developed by the China Meteorological Administration in accordance with the “basic data set of monthly report data files (A0/A1/A) after data correction of China's national ground stations in 1951–2010”, which is filed from the ground basic meteorological data construction project. After effective quality control, the availability rate of the meteorological factor data was generally more than 99%, and the accuracy rate was nearly 100%. This data included eight major items, including the daily air pressure values, air temperature, precipitation, evaporation, relative humidity, wind speed and direction, sunshine time, and 0 cm ground temperature. The data has been acquired daily from 824 basic meteorological stations located in China since January 1951.

In this study, precipitation, wind speed, and wind direction data were selected from the above data sets. In the precipitation data, there were ten columns of valid data, including station number, latitude, longitude, height of sea drawing in the observation field, year, month, day, accumulated precipitation from 20:00 of the previous day to 08:00, accumulated precipitation from 08:00 to 20:00, and accumulated precipitation from 20:00 of the previous day to 20:00. In the wind data, there were 12 columns of valid data, and these included the station number, latitude, longitude, height of sea drawing in the observation field, year, month, day, average wind speed, maximum wind speed, the direction of maximum wind speed, extreme wind speed, and the direction of the extreme wind speed.

Maximum wind speed refers to the average maximum wind speed over 10 min during a given period. Extreme wind speed refers to the maximum instantaneous wind speed within a given period. Under normal circumstances, the daily average wind speed was obtained by calculating the average of four values observed at 02:00, 08:00, 14:00, and 20:00. However, when the self-recording instrument was not equipped at the observation station, the daily average wind speed was the average of three wind speed values observed at 08:00, 14:00, and 20:00. The wind direction was expressed relative to 16 azimuths. The representation directions were N, NNE, NE, ENE, E, ESE, SE, SSE, S, SSW, SW, WSW, W, WNW, NW, and NNW. The absence of wind was also recorded.

**Figure 1:**
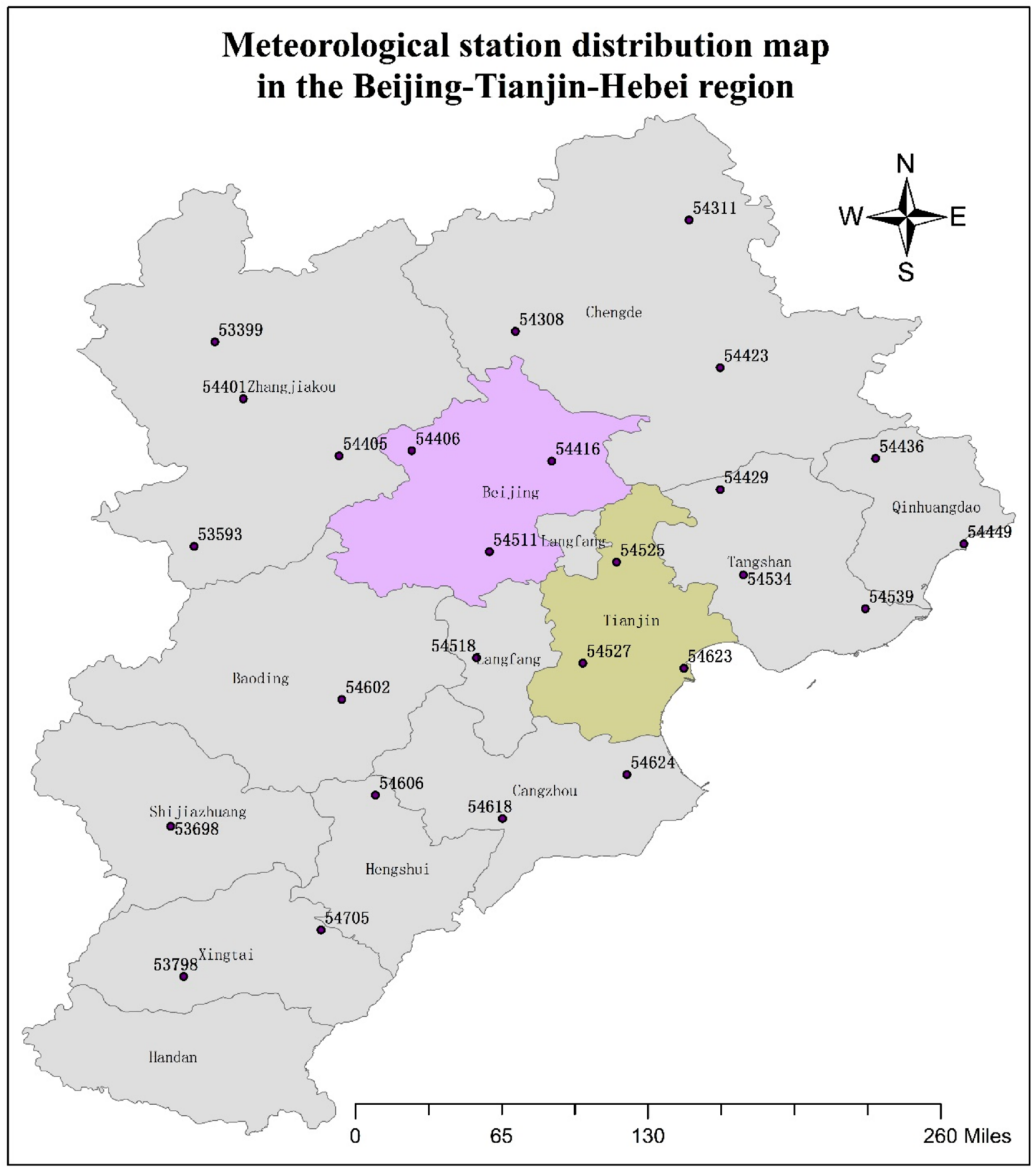
Meteorological station distribution map in the Beijing–Tianjin–Hebei region. The present map was done by using ArcGIS 10.0 desktop version (https://www.esri.com/zh-cn/arcgis/products/arcgis-pro/overview).

In the data sets, as there was no distinction between rainfall and snowfall, the precipitation over the whole winter period was used to represent snowfall for studying the statistical characteristics of snow and wind. In winter, the precipitation is mostly in the form of snowfalls in the Beijing–Tianjin–Hebei Region. The winter months are January, February, November, and December. After importing the above data into a database, the effective data from 26 observation stations in the Beijing–Tianjin–Hebei region was selected for this analysis. The location and number of the 26 stations are shown in Fig. 1. The selected time period was from 1968 to 2016 and included a total of 5893 days. During this period, due to force majeure, there was a lack of observation data for a portion of the time. However, the number of missing days at some sites was very small. The biggest lost count was 58 at station 54618. For this situation, the missing data was inferred based on the mean of the data measured before and after the gap.

## Results

### Wind scale distribution in winter

In this section, a method of generating the accumulative wind scale distribution map is proposed, and then, the characteristics of this map and the maximum wind direction for some stations are introduced in the Beijing–Tianjin–Hebei region in winter. In the field of meteorological forecasting, the wind scale of a certain day is divided into 18 classes according to the average wind speed. The basis of the classification is “Wind Scale”^21^. The specific classification method is shown in Table 1^21^. According to this method, the appearance frequency of different daily wind scales at 26 stations in the Beijing–Tianjin–Hebei region was extracted over the periods of 1, 2, 11, and 12 months from 1968 to 2016. The specific results are shown in Table 2.

**Table 1:** Wind scale classification.

Wind scale	Wind speed (m/s)	Wind scale	Wind speed (m/s)	Wind scale	Wind speed (m/s)
0	0.0–0.2	6	10.8–13.8	12	32.7–36.9
1	0.3–1.5	7	13.9–17.1	13	37.0–41.4
2	1.6–3.3	8	17.2–20.7	14	41.5–46.1
3	3.4–5.4	9	20.8–24.4	15	46.2–50.9
4	5.5–7.9	10	24.5–28.4	16	51.0–56.0
5	8.0–10.7	11	28.5–32.6	17	≥ 56.1

**Table 2:** Appearance frequency of different daily wind scales in the Beijing–Tianjin–Hebei region over the periods of 1, 2, 11, and 12 months from 1968 to 2016.

Station number	Province	Wind scale 0	Wind scale 1	Wind scale 2	Wind scale 3	Wind scale 4	Wind scale 5	Wind scale 6	Wind scale 7	Wind scale 8
54525	Tianjin	38	2021	2436	1000	302	71	9	0	0
54527	Tianjin	54	1967	2625	928	284	35	0	0	0
54623	Tianjin	22	674	2459	1816	677	200	44	1	0
54406	Beijing	97	2255	2241	942	274	54	7	4	0
54416	Beijing	18	1387	3403	951	127	2	0	0	0
54511	Beijing	23	1924	2639	968	274	55	10	0	0
53399	Hebei	14	640	2078	1861	943	269	66	8	1
53593	Hebei	104	3772	1684	301	29	2	0	0	0
53698	Hebei	56	3547	1953	262	72	2	1	0	0
53798	Hebei	48	3694	1916	207	26	2	0	0	0
54308	Hebei	255	2449	1860	942	326	56	5	0	0
54311	Hebei	170	2338	2475	802	106	2	0	0	0
54401	Hebei	25	1196	3081	1309	270	12	0	0	0
54405	Hebei	37	1620	1671	1374	927	238	25	1	0
54423	Hebei	1333	2887	1304	336	32	1	0	0	0
54429	Hebei	326	3663	1576	285	41	1	0	0	0
54436	Hebei	286	3778	1565	226	35	1	0	0	0
54449	Hebei	6	1432	3460	889	96	8	0	0	0
54518	Hebei	34	2535	2647	538	117	18	0	0	0
54534	Hebei	22	2004	3008	747	103	8	1	0	0
54539	Hebei	7	1115	3260	1166	302	36	7	0	0
54602	Hebei	40	3003	2478	353	18	1	0	0	0
54606	Hebei	61	2512	2833	453	30	1	0	0	0
54618	Hebei	26	1981	3055	674	94	5	0	0	0
54624	Hebei	3	905	3489	1262	207	22	2	1	0
54705	Hebei	38	2077	2950	734	84	10	0	0	0

**Figure 2:**
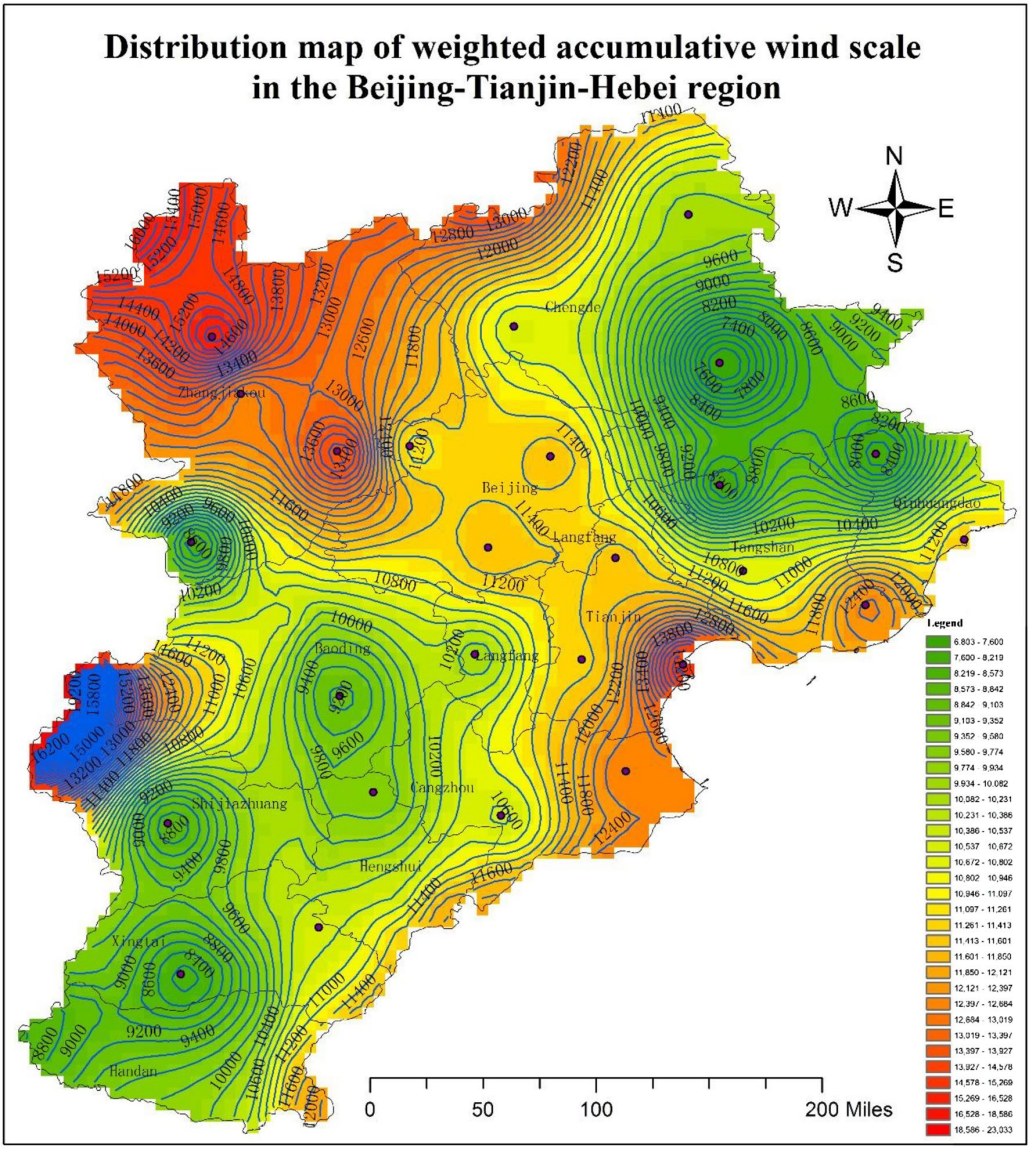
Distribution map of the weighted accumulative wind scale in the Beijing–Tianjin–Hebei region. The present map was done by using ArcGIS 10.0 desktop version (https://www.esri.com/zh-cn/arcgis/products/arcgis-pro/overview).

If we define wind scale 1 and 2 as small wind scale, wind scale 3, 4 and 5 as moderate wind scale, and scale 6, 7 and 8 as high wind scale, it can be seen in Table 2 that high wind scales frequently appeared at station 54623 of Tianjin city as well as station 53399 and 54405 of Hebei Province. As shown in Table 1, the wind speed at the different grades of wind scale was approximately equal to an arithmetic progression. For the convenience of making calculations, we multiplied the different wind scales and their corresponding appearance frequency for every station, and then carry on the accumulation. Based on this method, the accumulative wind scale distribution map can be obtained if the contemporaneous data from the nearby regional sites are added during the calculation process. Taking station 54525 in Tianjin city as an example, its valid data days were 5877. According to the valid data, its nominal accumulative wind grade was 0 × 38 + 1 × 2021 + 2 × 2436 + 3 × 1000 + 4 × 302 + 5 × 71 + 6 × 9 = 11,510, and its average wind scale was about 1.95848. Due to the absence of data over 16 days, the weighted cumulative wind scale calculated in this study was 11,510 + 1.95848 × 16 = 11,541.33568. After this calculation, the final distribution map representing the weighted accumulative wind scale was generated, as shown in Fig. 2.

As is shown in Fig. 2, in the Beijing–Tianjin–Hebei region, the weighted accumulative wind scale was larger in Tianjin, Beijing, and Zhangjiakou, while the other areas had a smaller accumulative wind scale. That is to say, in the Beijing–Tianjin–Hebei region, a wide ventilation corridor exists along Zhangjiakou, Beijing, and Tianjin. Because of the obstruction provided by the Taihang and Yanshan mountains, the wind from the north of the Inner Mongolia autonomous region can only move into the connection gap between these two major mountains and Zhangjiakou city, which causes strong winds throughout the year in Zhangjiakou city. At the same time, this wind blows along the main wind direction to Beijing city and gradually weakens. The wind from the sea passes through the gap between the Bohai Sea and Tianjin city and then enters the Beijing–Tianjin–Hebei region, which causes strong winds to occur in Tianjin city. This wind also blows along the main wind direction to Beijing city and gradually weakens. Due to the above two events, the wind scale in Beijing is relatively larger compared with the surrounding areas. Additionally, the wind scale of coastal areas (Qinhuangdao City, Tangshan City, and Cangzhou City) are larger than that of other inland areas, which means that the wind from the sea has an evident influence on the coastal areas.

**Figure 3:**
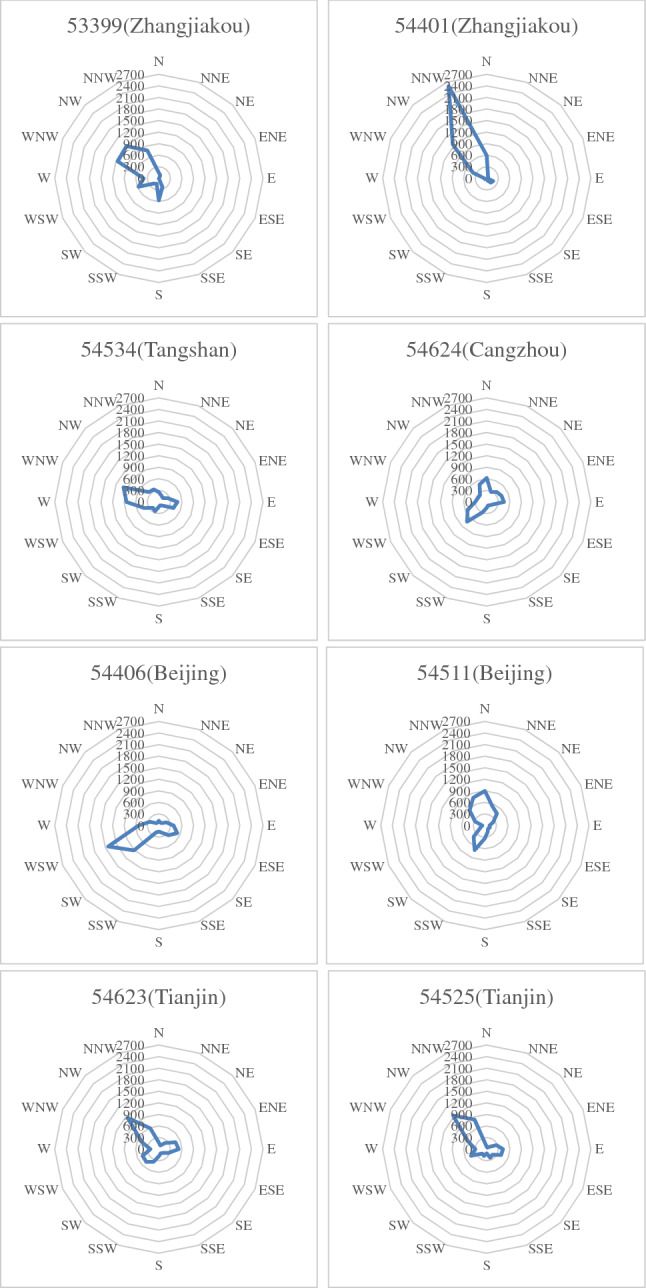
Maximum wind direction rose diagrams for some stations in the Beijing–Tianjin–Hebei region.

The maximum wind direction from all stations in the region was extracted from the study period, and this data was used to draw wind direction rose diagrams representing the maximum wind scale. The wind direction rose diagrams for some stations are shown in Fig. 3. As is shown in Fig. 3, it can be seen that a northwest wind prevails in Zhangjiakou city and Tianjin city in the winter, while east and west winds prevail in Tangshan city and Cangzhou City. These observations indicated that the wind from the northwest area and the wind from the sea could affect this region.

### Basic wind speed in winter

In this section, a method of calculating the basic wind speed is introduced, and then, the basic wind speed distribution map is generated and analyzed in the Beijing–Tianjin–Hebei region in winter. In the design specification of wind load on buildings, the reoccurrence period of wind load for general structures in China is set as 50 years. Therefore, it is important to calculate the reoccurrence period of wind and snow accurately. As a consequence, the annual maximum wind speed in winter during the 49-year period was extracted. For historical reasons, this data in the years 1968, 1969, and 1970 for all of the stations in this region are missing. In addition, the data in the year 1971 for station 54618 and for the years 1971 and 1972 for station 54606 are also absent.

Based on the above available data, using a Gumbel curve with an extreme type I distribution, the basic wind speed in the reoccurrence period of 50 and 100 years was calculated. The calculation steps are as follows.

First, the average maximum wind speed $\overline{v}_{i}$ and its root variance $\sigma_{i}$ within a specified period of each station were calculated. The calculation formula $\overline{v}_{i}$ for station number $i$ is shown in Eq. (1). The formula $\sigma_{i}$ for station number $i$ is shown in Eq. (2). $v_{ij}$ is the maximum wind speed for station number $i$ in the winter of the year $j$, and $n$ is the year from 1971 to 2016 for most stations

1 $$\overline{v}_{i} = \sum\limits_{j = 1}^{n} {v_{ij} }$$

2 $$\sigma_{i} = \sqrt {\frac{{\sum\nolimits_{j = 1}^{n} {(v_{ij} - \overline{v}_{i} )^{2} } }}{n - 1}}$$

Second, the assurance rate $P$ (according to the recurrence period $T$) was obtained. The calculation formula for $P$ is shown in Eq. (3). The value of $T$ was set to 50 and 100 years

3 $$P = 1 - \frac{1}{T}$$

Based on the assurance rate $P$, the assurance coefficient $\varphi$ can be further obtained according to the reference table presenting the assurance coefficient of the Gumbel curve with an extreme type I distribution, which is shown in Table 3.

**Table 3:** Reference table of the assurance coefficient of the Gumbel curve with an extreme type I distribution.

$P$	0.60	0.70	0.80	0.90	0.95	0.97	0.98	0.99	0.995	0.999	0.9995	0.9999
$\varphi$	0.07	0.35	0.72	1.30	1.87	2.27	2.59	3.14	3.68	4.94	5.48	6.73

Finally, formula (4) was adopted to calculate the basic wind speed at each station during the different reoccurrence periods. The calculated results are shown in Table 4

4 $$V_{i} = \overline{v}_{i} + \sigma_{i} \cdot \varphi$$

**Table 4:** Basic wind speed of each station in the Beijing–Tianjin–Hebei region at different reoccurrence periods.

Station ID	Province	Basic wind speed in 50 years	Corresponding wind scale in 50 years	Basic wind speed in 100 years	Corresponding wind scale in 100 years
54525	Tianjin	20.45	8	21.84	9
54527	Tianjin	23.13	9	25.06	10
54623	Tianjin	26.88	10	29.14	11
54406	Beijing	17.3	8	18.52	8
54416	Beijing	16.29	7	17.46	8
54511	Beijing	21.34	9	23.07	9
53399	Hebei	25.81	10	27.79	10
53593	Hebei	13.9	7	14.62	7
53698	Hebei	16.54	7	17.84	8
53798	Hebei	16.03	7	17.44	8
54308	Hebei	19.49	8	20.85	9
54311	Hebei	14.28	7	15.14	7
54401	Hebei	15.87	7	16.91	7
54405	Hebei	18.76	8	19.74	8
54423	Hebei	17.21	8	18.57	8
54429	Hebei	18.88	8	20.59	8
54436	Hebei	13.12	6	13.99	7
54449	Hebei	15.13	7	16.18	7
54518	Hebei	19.77	8	21.48	9
54534	Hebei	19.04	8	20.53	8
54539	Hebei	20.28	8	21.87	9
54602	Hebei	15.2	7	16.29	7
54606	Hebei	15.58	7	16.62	7
54618	Hebei	19.16	8	20.73	8
54624	Hebei	19.18	8	20.46	8
54705	Hebei	20.66	8	22.39	9

**Figure 4:**
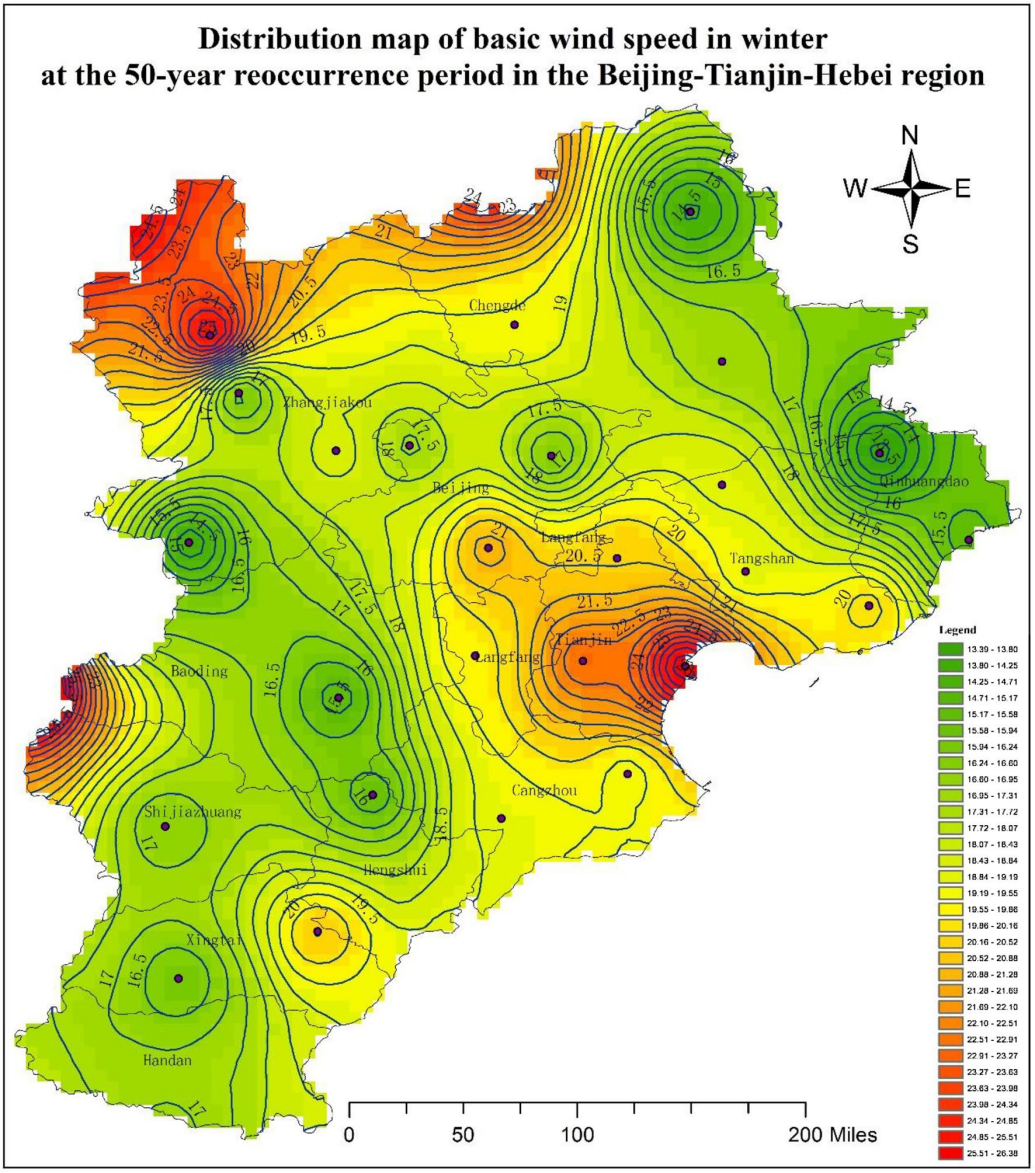
Distribution map of the basic wind speed in winter at the 50-year reoccurrence period in the Beijing–Tianjin–Hebei region. The present map was done by using ArcGIS 10.0 desktop version (https://www.esri.com/zh-cn/arcgis/products/arcgis-pro/overview).

Based on the results presented in Table 4, a distribution map of the basic wind speed in winter at the 50-year reoccurrence period in the Beijing–Tianjin–Hebei region was generated, as shown in Fig. 4. This analysis indicated that the wind speed was large in the north of Zhangjiakou, northwest of Chengde, Tianjin, Langfang, the junction of Xingtai and Hengshui, and the west of the junction of Shijiazhuang and Baoding. The wind speed was low in Qinghuangdao, the east of Chengde, the southwest of Zhangjiakou, the north of Beijing, Baoding, the north of Hengshui, the east of Shijiazhuang, the west of Xingtai, and Handan. Consequently, these results can be referred to the code of building structure design in the Beijing–Tianjin–Hebei region.

### Snowfall distribution in winter

In this section, a method of generating the accumulative snowfall distribution map is proposed, and then, the characteristics of this map are introduced in the Beijing–Tianjin–Hebei region in winter. In winter, precipitation is usually represented as snowfall. There is strict regulation about snowfall grade standard in meteorology. Snowfall is the depth of an equal amount of water, which is turned from snow. Like rainfall, snowfall refers to the amount of snow that falls within a certain period, and this is generally measured over 24 h. According to the standard “Short-range Weather Forecast”^22^, snowfall is divided into seven grades: sporadic light snow, light snow, moderate snow, heavy snow, blizzard, storm snow, and super-big blizzard^22^. If the snowfall is much bigger than 10 mm, the grade super-big blizzard can be sub-divided into two levels: big blizzard and super-big blizzard.

The defined snowfall occurring at each grade within 24 h is shown in Table 5. As the occurrence of sporadic light snow was very small, the sporadic light snow and light snow data were merged. Subsequently, the times of the different snowfall grades at the 26 stations in the Beijing–Tianjin–Hebei region within 1, 2, 11, and 12 months from 1968 to 2016 were extracted. The results are shown in Table 6.

**Table 5:** Snowfall grade classification.

Snowfall grade	24 h of precipitation (mm)
Sporadic light snow	≤ 0.1
Light snow	0.1–2.4
Moderate snow	2.5–4.9
Heavy snow	5.0–9.9
Blizzard	10.0–19.9
Storm snow	20.0–30.0
Super-big blizzard	> 30.0

**Table 6:** Occurrence times of different snowfall grades in the Beijing–Tianjin–Hebei region within 1, 2, 11, and 12 months from 1968 to 2016.

Station number	Provinces	Times of light snow	Times of moderate snow	Times of heavy snow	Times of blizzard	Times of storm snow	Times of super-big blizzard
54525	Tianjin	214	52	41	15	4	1
54527	Tianjin	260	48	43	16	3	0
54623	Tianjin	268	69	32	21	4	0
54406	Beijing	234	52	24	10	1	1
54416	Beijing	237	54	34	15	1	1
54511	Beijing	280	38	40	8	4	1
53399	Hebei	502	33	11	3	1	0
53593	Hebei	340	42	17	9	2	0
53698	Hebei	282	69	49	24	1	3
53798	Hebei	309	67	45	21	5	1
54308	Hebei	233	36	20	1	1	1
54311	Hebei	316	41	15	2	0	0
54401	Hebei	265	53	21	5	1	0
54405	Hebei	228	44	18	3	1	1
54423	Hebei	232	42	33	8	0	0
54429	Hebei	307	58	30	12	3	1
54436	Hebei	307	52	25	10	3	0
54449	Hebei	277	55	32	16	3	2
54518	Hebei	237	50	29	17	2	1
54534	Hebei	282	61	36	19	1	1
54539	Hebei	295	64	44	16	2	1
54602	Hebei	248	45	27	20	3	0
54606	Hebei	280	56	45	18	3	0
54618	Hebei	272	50	46	17	6	0
54624	Hebei	284	59	45	27	3	0
54705	Hebei	313	69	50	25	4	0

As can be seen in Table 6, the times of high snowfall at stations 53399, 53593, 54308, 54311, 54401, 54405, and 54423 in Hebei were relatively less compared to the other stations. According to the snowfall amount at each snowfall grade over 24 h, the grades were standardized: light snow, 1; moderate snow, 2; heavy snow, 4; blizzard, 8; storm snow, 12; and super-big blizzard, 16.

**Figure 5:**
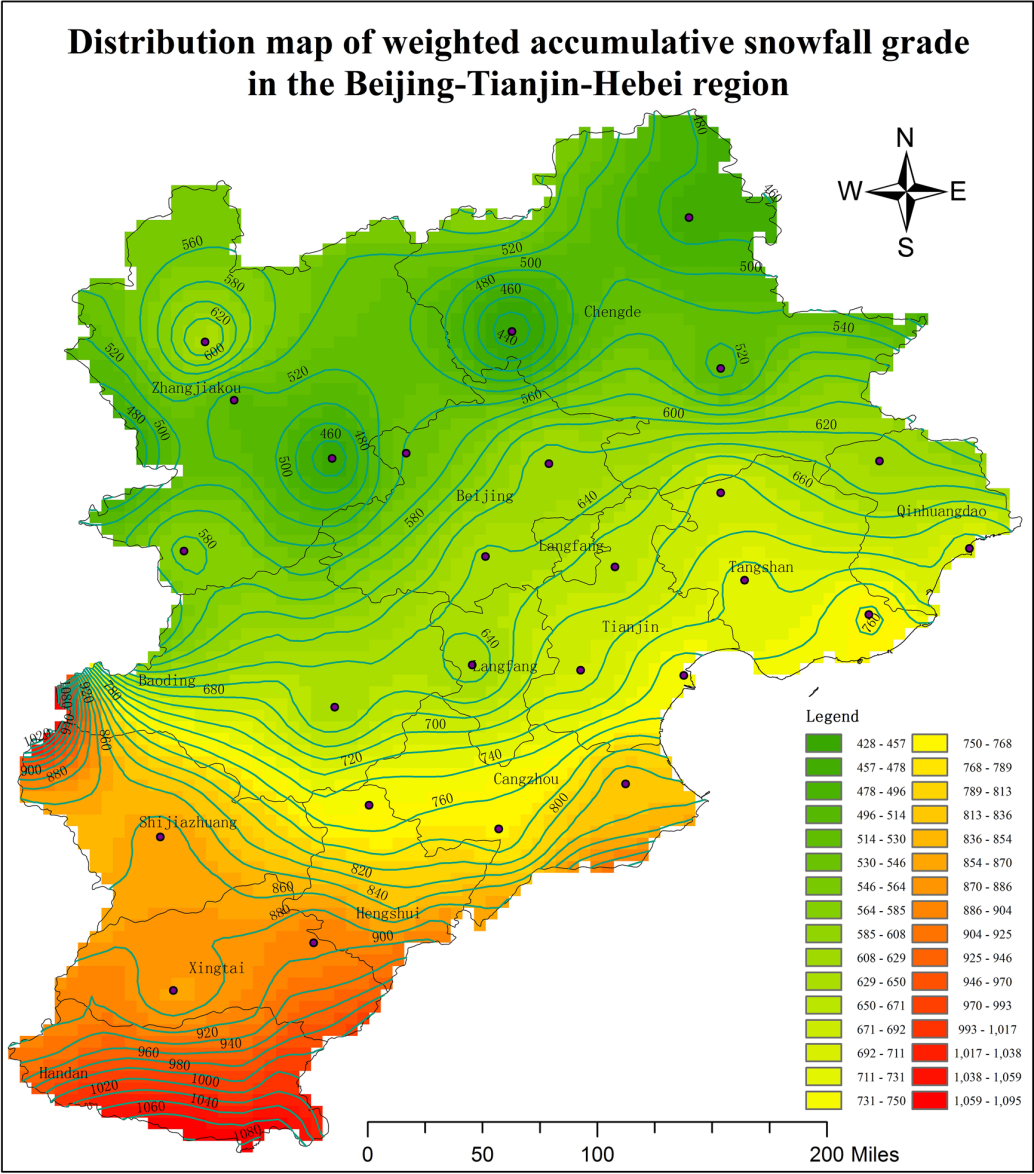
Distribution map of weighted accumulative snowfall grade in the Beijing–Tianjin–Hebei region. The present map was done by using ArcGIS 10.0 desktop version (https://www.esri.com/zh-cn/arcgis/products/arcgis-pro/overview).

As an example, at station 54525 in Tianjin, the weighted cumulative snowfall grade was 214 × 1 + 52 × 2 + 41 × 4 + 15 × 8 + 4 × 12 + 1 × 16 = 666. Based on this method, the weighted accumulative snowfall grade for all of the stations in the Beijing–Tianjin–Hebei region and nearby was calculated, and the distribution map of the weighted cumulative snowfall grade in the Beijing–Tianjin–Hebei region was generated, as shown in Fig. 5.

As can be seen in Fig. 5, the snowfall in the Beijing–Tianjin–Hebei region could be divided into three zones. The heavy snowfall area included Shijiazhuang, Xingtai, and Handan. The moderate snowfall area included Hengshui, Cangzhou, Baoding, Tianjin, Beijing, Langfang, Tangshan, and Qinhuangdao. The light snowfall area included Zhangjiakou and Chengde. Based on these observations, the snowfall was roughly in line with the occurrence of heavy snowfall in the southern region, moderate snowfall in the central region, and light snowfall in the northern region. Additionally, the boundary between the heavy snowfall area and moderate snowfall area could be defined as the connection between the northern part of Shijiazhuang City and the central part of Hengshui City, indicating that the boundary between the different snowfall areas was not strictly based on latitude. The boundary between the moderate snowfall area and light snowfall area could be defined as the connection between Baoding city, the northwest part of Beijing City, and the south part of Chengde City.

### Cumulative snowfall and wind scale in relation to the top ten maximum snowfall events in winter

In this section, a method of generating the cumulative snowfall and wind scale distribution map in relation to the top ten maximum snowfall events in winter is proposed, and then, the characteristics of this map are introduced and analyzed in the Beijing–Tianjin–Hebei region. As the occurrence of snowdrift formation requires heavy snowfall and strong winds to appear at the same time, the top-ten biggest snowfalls at all of the stations in the Beijing–Tianjin–Hebei region from the year 1965 to 2016 were first extracted, and the values of wind scale for each day in this period, from the first day of snowing to 5 days after the day the snow had stopped, was then extracted. Ultimately, the above snowfalls and wind scales were summed by station prior to the statistical analysis. Based on this method, the accumulative amount of snowfall, total snow days, average daily snowfall, accumulative wind scale, total wind days, and average daily wind scale were calculated for each station. Among these wind data, one day of data for stations 53399, 53593, and 54436 in Hebei were missing, and three days of data for station 54525 in Tianjin were missing. In order to account for the missing data, the weighted cumulative wind scale was obtained using the method detailed above. The processing for these stations was the same as that described in “Conclusions and discussions”. To facilitate comparisons between the wind and snowfall numbers, the accumulative amount of snowfall and accumulative wind scale data were normalized. The final statistical results are presented in Table 7.

**Table 7:** Accumulative snowfall and accumulative wind scale taken from the top ten maximum snowfall events at each station in the Beijing–Tianjin–Hebei region.

Station number	Province	Accumulative amount of snowfall (mm)	Total snowing days	Accumulative wind scale	Total windy days	Normalized accumulative snowfall	Normalized accumulative wind scale	Average daily snowfall (mm)	Average daily wind scale
53399	Hebei	130.2	29	176	79	0.435	0.931	4.490	2.228
53593	Hebei	193.3	25	107.13	75	0.646	0.567	7.732	1.428
53698	Hebei	231.7	32	109	82	0.775	0.577	7.241	1.329
53798	Hebei	259.1	32	129	82	0.867	0.683	8.097	1.573
54308	Hebei	168.3	27	155	77	0.563	0.820	6.233	2.013
54311	Hebei	95	25	109	75	0.318	0.577	3.800	1.453
54401	Hebei	116.4	20	144	70	0.389	0.762	5.820	2.057
54405	Hebei	151.5	25	169	75	0.507	0.894	6.060	2.253
54406	Beijing	221.1	24	118	74	0.739	0.624	9.213	1.595
54416	Beijing	211.2	24	134	74	0.706	0.709	8.800	1.811
54423	Hebei	151.7	26	93	76	0.507	0.492	5.835	1.224
54429	Hebei	237.3	25	108	75	0.794	0.571	9.492	1.440
54436	Hebei	210.3	21	106.43	71	0.703	0.563	10.014	1.499
54449	Hebei	299	28	153	78	1.000	0.810	10.679	1.962
54511	Beijing	224.5	26	157	76	0.751	0.831	8.635	2.066
54518	Hebei	221.7	29	130	79	0.741	0.688	7.645	1.646
54525	Tianjin	248.3	25	166.73	75	0.830	0.882	9.932	2.223
54527	Tianjin	222.3	24	175	74	0.743	0.926	9.263	2.365
54534	Hebei	236	27	159	77	0.789	0.841	8.741	2.065
54539	Hebei	229.1	27	171	77	0.766	0.905	8.485	2.221
54602	Hebei	226.3	32	143	82	0.757	0.757	7.072	1.744
54606	Hebei	233	27	131	77	0.779	0.693	8.630	1.701
54618	Hebei	221.7	28	140	78	0.741	0.741	7.918	1.795
54623	Tianjin	234.3	23	185	73	0.784	0.979	10.187	2.534
54624	Hebei	267.3	32	189	82	0.894	1.000	8.353	2.305
54705	Hebei	236.6	29	143	79	0.791	0.757	8.159	1.810

**Figure 6:**
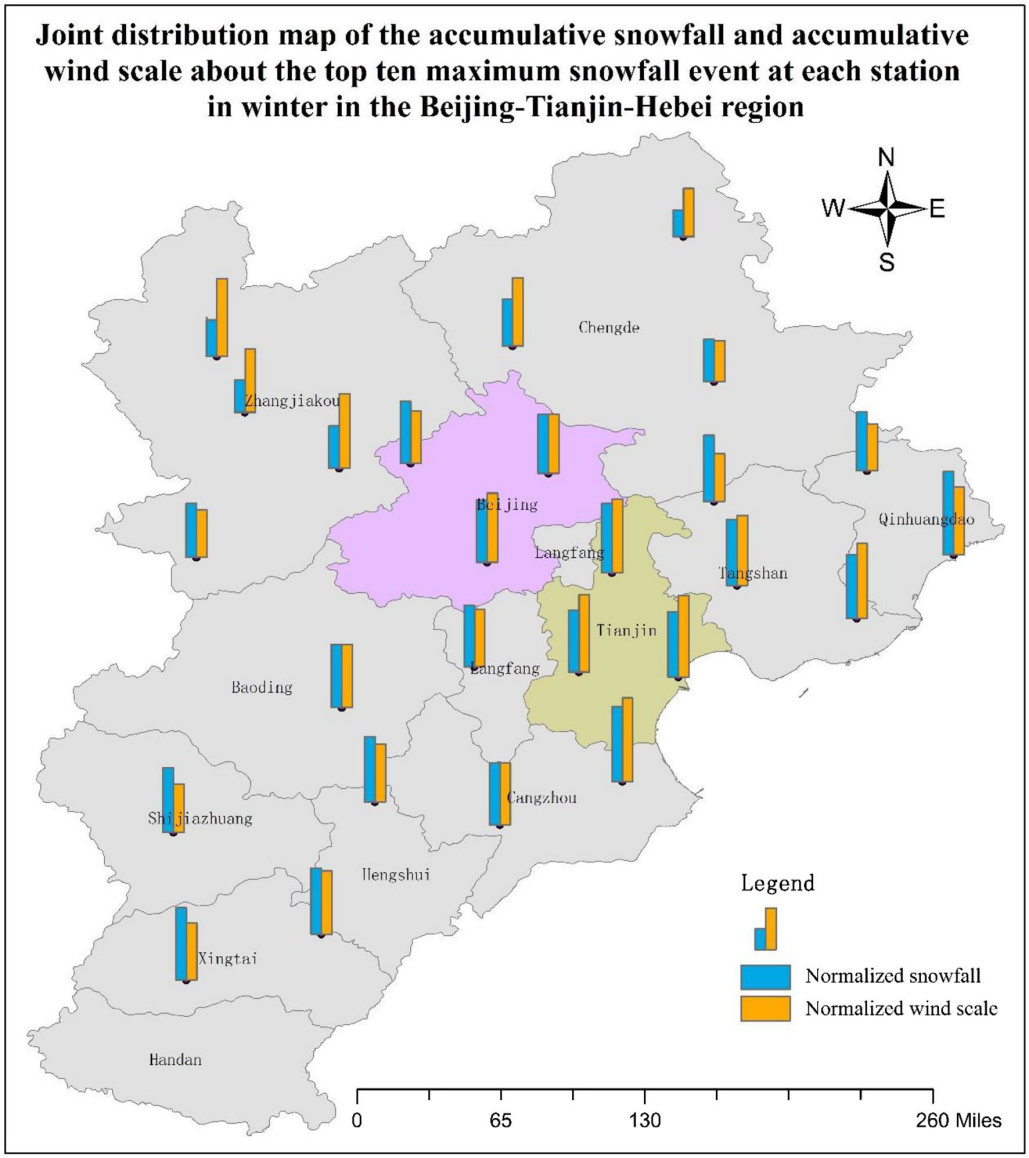
Joint distribution map of the accumulative snowfall and accumulative wind scale from the top ten maximum snowfall events at each station in the Beijing–Tianjin–Hebei region. The present map was done by using ArcGIS 10.0 desktop version (https://www.esri.com/zh-cn/arcgis/products/arcgis-pro/overview).

Based on the normalized snowfall and normalized wind scale data, a joint distribution map of the accumulative snowfall and accumulative wind scale from the top ten maximum snowfall events at each station in the Beijing–Tianjin–Hebei region was generated (Fig. 6). It can be seen from this analysis that the wind scale and snowfall values in Tianjin, Tangshan, Cangzhou, Qinhuangdao, and Langfang were large, and each of these areas is proximal to the Bohai Sea. The wind and snowfall values were medium in Baoding and Hengshui, and the snowfall amount was larger and the wind value was smaller in Shijiazhuang. In Zhangjiakou, the wind value was large, but the snowfall amount was small. Both the wind and snowfall values in Chengde were small.

## Conclusions and discussions

This study assessed the spatial distribution characteristics of wind and snowfall in the Beijing–Tianjin–Hebei region of China based on the “Daily Data Sets of Climate Data for China International Surface Exchange Station (V3.0)”. The following conclusions can be drawn from this analysis.

1. The accumulative wind scale is larger in Tianjin, Beijing, and Zhangjiakou and smaller in the other areas of the Beijing–Tianjin–Hebei region. In the Beijing–Tianjin–Hebei region, there is a wide ventilation corridor along Zhangjiakou, Beijing, and Tianjin. The wind scale in coastal areas (Qinhuangdao, Tangshan, and Cangzhou) is larger than that in the other inland areas, which also indicates that wind coming from the sea has a certain influence on the coastal areas.
2. Based on the distribution map depicting the basic wind speed in winter over the 49 years assessed in the Beijing–Tianjin–Hebei region, the conclusions are as follows: the wind speed is large in the north of Zhangjiakou, northwest of Chengde, Tianjin, Langfang, the junction of Xingtai and Hengshui, and west of the junction of Shijiazhuang and Baoding. The wind speed is low in Qinghuangdao, the east of Chengde, the southwest of Zhangjiakou, the north of Beijing, Baoding, the north of Hengshui, the east of Shijiazhuang, the west of Xingtai, and Handan.
3. The Beijing–Tianjin–Hebei region can be divided into three zones according to the snowfall amount occurring in winter. The heavy snowfall areas include Shijiazhuang, Xingtai, and Handan. The moderate snowfall areas include Hengshui, Cangzhou, Baoding, Tianjin, Beijing, Langfang, Tangshan, and Qinhuangdao. The light snowfall areas include Zhangjiakou and Chengde.
4. A methodology to extract the joint combination of wind scale and snowfall was proposed. The joint distribution map of the accumulative snowfall and accumulative wind scale for the top ten maximum snowfall events at each station in winter in the Beijing–Tianjin–Hebei region was generated based on these data. The wind scale and snowfall values in Tianjin, Tangshan, Cangzhou, Qinhuangdao, and Langfang are large, and these areas are proximal to the Bohai Sea. The snowfall amount is larger and the wind speed is lower in Shijiazhuang. In Zhangjiakou, the wind speed is high, but the snowfall amount is low. Both the wind speed and snowfall amounts in Chengde are low.

The above results and conclusions have important practical significance for the design of building structures in the Beijing–Tianjin–Hebei region. When we calculate the snow and wind load for a building structure in this region, the current method is that: first, query the basic snow pressure and the basic wind pressure in this region from the *Load code for the design of building structures of China*^7^, second, calculate the snow and wind load marked by $L_{snow}$ and $L_{windO}$, respectively, third, add two numbers together and get the total load of snow and wind, which is marked by $L_{snow + windO}$. Actually the total load calculated by this method is bigger than the true value. That is the problem which is solved in this study. For the same building structure in this region, the snow load is still expressed by $L_{snow}$, and the temp wind pressure after the biggest snowfall events should be calculated according to this study, which is marked by $L_{windT}$, and then, the joint load of snow and wind can be gotten by adding the two numbers $L_{snow}$ and $L_{windT}$ together, which is marked by $L_{snow + windT}$. The value of $L_{snow + windT}$ should be bigger than the value of $L_{snow}$, and smaller than the value of $L_{snow + windO}$. If the value of $L_{snow + windT}$ is bigger than the value of $L_{windO}$, the total load of snow and wind should be the value of $L_{snow + windT}$, not the value of $L_{snow + windO}$. If the value of $L_{snow + windT}$ is smaller than the value of $L_{windO}$, the total load should be the value of $L_{windO}$. In this study, we just studied statistical characteristics of the spatial distribution of wind and snowfall in the Beijing–Tianjin–Hebei region of China. In the future, their spatial distributive characteristics in more areas will be surveyed and analyzed.

## Data Availability

All meteorological data associated with this manuscript are available and can be found here: http://data.cma.cn/data/cdcdetail/dataCode/SURF_CLI_CHN_MUL_DAY_V3.0.html. The administrative areas of the Beijing–Tianjin–Hebei region of China are freely available for academic use and other non-commercial use. Using the data to create maps for academic publishing is allowed in the license. The data can be found here: https://gadm.org/data.html.
